# Caught in a Cocoon: Operative Release of Primary Sclerosing Encapsulating Peritonitis With Endoscopic Management of Early Postoperative Duodenojejunal Intussusception

**DOI:** 10.7759/cureus.109212

**Published:** 2026-05-19

**Authors:** Chun Pui Joshua Wong, Sajid Malik, Samina Alim, Parth Paskaran, Francesco DiMaggio

**Affiliations:** 1 General Surgery, Croydon University Hospital, London, GBR; 2 General Surgery, Croydon Health Services NHS Trust, Croydon, GBR; 3 Gastroenterology, Croydon University Hospital, London, GBR

**Keywords:** duodenojejunal flexure, duodenojejunal intussusception, endoscopic pneumatic decompression, sclerosing encapsulating peritonitis (sep), small-bowel obstruction, surgical case reports

## Abstract

Sclerosing encapsulating peritonitis (SEP) is a rare cause of small bowel obstruction in which a fibrocollagenous membrane encases the small bowel. Preoperative diagnosis is often difficult because computed tomography (CT) findings may be subtle or atypical. This report highlights a focal proximal jejunal phenotype misinterpreted as an internal hernia on CT and demonstrates a multidisciplinary approach that achieved complete resolution. A 36-year-old man presented with 7-8 weeks of progressive upper abdominal pain, daily bilious vomiting, constipation, and weight loss, with a history of similar self-limiting episodes. CT demonstrated marked gastric and proximal duodenal distension with a distal duodenal transition point and an apparent proximal jejunal abnormality, initially interpreted as a possible internal hernia. Laparotomy identified a dense encapsulating membrane involving the small bowel from the duodenojejunal (DJ) flexure to the mid-jejunum; membrane division and adhesiolysis were performed without bowel resection. Histopathology of the excised membrane demonstrated hyalinized fibrous tissue with mild inflammatory change, without granulomas or malignancy, supporting a diagnosis of idiopathic non-granulomatous encapsulating peritonitis. Postoperatively, persistent proximal obstruction was associated with a short segment of residual DJ intussusception, which was managed successfully with endoscopic pneumatic decompression. At the 28-week email follow-up, the patient reported occasional mild postprandial abdominal discomfort but no frank vomiting, recurrent obstruction, or significant functional impairment. This case illustrates an important diagnostic pitfall: a proximal DJ transition point labeled as an internal hernia (or proximal jejunal cyst) may instead represent focal SEP, particularly in patients with recurrent episodes and no history of prior abdominal surgery. It also reinforces operative principles of complete membrane release and adhesiolysis with bowel preservation whenever possible. Persistent postoperative obstruction despite surgical release may reflect discrete mechanical complications. In clinically stable patients, CT findings can guide decision-making and may permit non-operative or endoscopic management. SEP may present with a proximal jejunal phenotype that mimics an internal hernia on CT and is often confirmed only intraoperatively. Early postoperative obstruction may require individualized multidisciplinary escalation based on clinical status and imaging findings.

## Introduction

Sclerosing encapsulating peritonitis (SEP), also known as abdominal cocoon syndrome, is a rare chronic inflammatory disorder characterized by a fibrocollagenous membrane encasing the small intestine, leading to bowel fixation, kinking, and obstruction [1-5]. SEP is classified as either primary (idiopathic) or secondary. The secondary form is associated with conditions that promote chronic peritoneal inflammation, including long-term peritoneal dialysis, abdominal tuberculosis, prior surgery, chronic beta-blocker use, cirrhosis, and intra-abdominal malignancy [1-7]. Due to its rarity, diagnosis is frequently delayed and, in many cases, is established only intraoperatively [1,3-5].

Contrast-enhanced computed tomography (CT) is the most informative preoperative imaging modality [1-6]. Characteristic findings include peritoneal thickening with enhancement, occasionally with calcification, and clustering of small-bowel loops encased by a soft-tissue-density membrane, described as a cocoon or cauliflower configuration [1-6]. Despite this, CT diagnosis remains challenging. In focal proximal presentations, imaging appearances may mimic internal herniation or other causes of small bowel obstruction, representing a specific diagnostic pitfall that can delay definitive management [2-4,6]. To our knowledge, there are no widely adopted consensus guidelines for the management of primary SEP.

This case describes a focal proximal phenotype centered at the duodenojejunal (DJ) flexure and proximal jejunum. CT findings were dominated by a jejunal transition point and were initially interpreted as an internal hernia. This anatomical pattern represents a distinct diagnostic pitfall, particularly in patients without prior abdominal surgery. Furthermore, the postoperative course illustrates that persistent obstruction following membrane release may reflect a discrete mechanical complication rather than recurrent encapsulation, supporting a structured multidisciplinary escalation strategy that includes endoscopic intervention.

We report a case of primary SEP presenting with recurrent subacute obstruction culminating in high-grade small bowel obstruction, managed surgically and endoscopically. This case provides practical operative and postoperative decision-making insights relevant to emergency general surgery and upper gastrointestinal practice in a district general hospital setting in the United Kingdom.

## Case presentation

A 36-year-old Asian male patient presented to the emergency department with a seven-to-eight-week history of progressively worsening upper abdominal pain, daily bilious vomiting, severe constipation, and significant unintentional weight loss. He reported an estimated weight loss of 8-10 kg during this period, associated with marked anorexia. His last bowel movement had occurred several weeks prior to presentation.

The patient had no significant past medical history and was otherwise fit and well. He had no history of previous abdominal surgery, peritoneal dialysis, chronic liver disease, malignancy, or autoimmune disease, and was not taking any regular medications. There was a remote history of residence in a tuberculosis-endemic region many years earlier, with no known contact with active tuberculosis and no recent travel within the preceding three years. There was no personal or family history of inflammatory bowel disease.

On examination, he was hemodynamically stable, with a body mass index (BMI) of 20.75 kg/m². Abdominal examination revealed a firm, non-tender, non-pulsatile fullness in the epigastric and left hypochondrial regions. The size was not formally measured clinically because the margins were difficult to define on palpation; however, this corresponded anatomically to the abnormality subsequently identified on CT imaging. There were no signs of peritonitis. Bowel sounds were normal and active, and no abdominal wall hernia was identified. The remainder of the systemic examination was unremarkable.

Admission blood tests demonstrated a hemoglobin of 175 g/L, white cell count of 10.1 × 10⁹/L, and platelet count of 264 × 10⁹/L. Renal function was within normal limits, with a sodium of 137 mmol/L, potassium of 4.0 mmol/L, urea of 6.0 mmol/L, and creatinine of 79 µmol/L. Albumin was 42 g/L. C-reactive protein (CRP) was elevated at 117 mg/L. Liver function tests demonstrated a mildly elevated alkaline phosphatase level of 124 U/L. Lipase was mildly elevated at 81 U/L. Venous blood gas analysis demonstrated a pH of 7.384 with a lactate of 1.5 mmol/L, indicating no evidence of tissue hypoperfusion. Blood glucose was mildly elevated at 6.2 mmol/L, likely reflecting a physiological stress response. Coagulation profile was normal.

He had experienced similar short-lived episodes over the preceding two years, characterized by abdominal pain and obstructive symptoms, resulting in multiple hospital presentations. On previous occasions, symptoms resolved spontaneously with conservative management, and no unifying diagnosis was established. Blood results during these admissions were largely unremarkable, with only occasional mild elevations in inflammatory markers, including a CRP of 16 mg/L and white cell count of 12.3 × 10⁹/L, consistent with a self-limiting inflammatory process. Following these episodes, he described intermittent abdominal discomfort but remained functionally independent until the current presentation.

Subsequent autoimmune and infective screening did not identify a clear secondary cause. Antinuclear, mitochondrial, smooth muscle, and liver microsomal antibodies were negative. A weakly positive perinuclear anti-neutrophil cytoplasmic antibody (pANCA) was noted; however, proteinase-3 and myeloperoxidase antibodies were normal, making clinically significant ANCA-associated vasculitis unlikely. *Cytomegalovirus *(CMV) IgG and hepatitis E IgG positivity were consistent with previous exposure, while human immunodeficiency virus (HIV), hepatitis B surface antigen, and hepatitis C antibody tests were negative.

The case was discussed with the upper gastrointestinal surgical team, and cross-sectional imaging with contrast-enhanced CT was requested. The stomach and proximal duodenum were markedly distended (Figures 1a, 1b). The jejunum demonstrated a markedly abnormal appearance, characterized by a 13 × 7 × 6 cm low-attenuation hypodense collection along the antimesenteric border, measuring approximately 13 Hounsfield units, consistent with fluid attenuation (Figure 1b). A transition point was identified at the proximal jejunum, with collapse distal to this point (Figures 1b, 1c). The downstream small and large bowel appeared unremarkable. There was no free intra-abdominal fluid or gas, and the solid organs appeared normal. No lymphadenopathy was identified. Overall, the appearances were interpreted as focal jejunal pathology (Figure 1), with differential diagnoses including a large cystic lesion or an internal hernia causing high-grade obstruction. The decision was made to proceed with emergency laparotomy.

**Figure 1:**
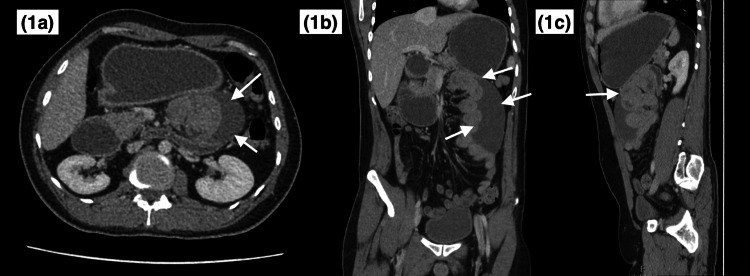
Preoperative computed tomography images (a) Marked dilatation of the stomach and duodenum. The jejunum demonstrates an unusual appearance, with a 13 × 7 × 6 cm low-attenuation collection along the antimesenteric border. (b) Jejunal transition point with collapse of the distal bowel loops. (c) Small intestine encapsulated within a “cocoon” appearance.

Emergency laparotomy was performed within 48 hours of admission. During this interval, the patient was managed with nasogastric decompression using four-hourly aspirates, intravenous crystalloid resuscitation, sips of water, and initiation of a proton pump inhibitor. A peripherally inserted central catheter (PICC) line for parenteral nutrition had been planned but was not inserted prior to surgery. He therefore proceeded to theater in a nutritionally depleted state, having received no formal preoperative nutritional support. Total parenteral nutrition was commenced postoperatively once central venous access had been established.

Given the patient’s remote history of residence in a tuberculosis-endemic region, tuberculosis was considered a potential secondary cause of peritoneal inflammation. A QuantiFERON-TB enzyme-linked immunosorbent assay (ELISA) test was sent and returned negative. There was no history of known tuberculosis contact or recent international travel within the preceding three years. The patient had no cough and was unable to produce sputum. Chest radiography demonstrated minimal bibasal atelectatic change without evidence of an acute pulmonary lesion. CT chest demonstrated patchy consolidation and tree-in-bud opacities in the right mid-to-lower zones, with no mediastinal, hilar, or supraclavicular lymphadenopathy and no destructive osseous lesions. These findings were considered most consistent with early infective or inflammatory change. Given the high-grade obstruction and clinical deterioration, surgery was not delayed pending completion of the tuberculosis workup. Postoperative multidisciplinary review involving microbiology and respiratory teams was subsequently undertaken to further exclude tuberculosis as an underlying etiology.

Therapeutic intervention

A midline laparotomy was performed via a xipho-infraumbilical incision. On entry into the peritoneal cavity, extensive adhesions were encountered involving the small bowel mesentery and the parietal peritoneum.

A dense, thick fibrocollagenous capsule was identified encasing the small bowel from the DJ flexure to the mid-jejunum. The capsule appeared continuous with the parietal peritoneum and resulted in multiple levels of small bowel obstruction. A clear transition point was noted approximately 80 cm distal to the DJ flexure, where the encapsulating membrane ended and the distal small bowel appeared collapsed.

The fibrocollagenous membrane encasing the bowel was incised longitudinally along the antimesenteric border at multiple levels. The incision was made through the extrinsic capsule rather than the bowel serosa, which was intact on inspection. This maneuver released the constricted small-bowel loops and allowed them to regain length and mobility. Adhesions along the mesenteric aspect were then carefully divided, and final inspection confirmed viable bowel with no serosal breach, perforation, or intrinsic lesion.

Following the release of the encapsulating membrane, the small intestine was systematically examined from the DJ flexure to the ileocecal valve. The preoperatively palpable epigastric/left hypochondrial mass corresponded to tightly clustered proximal jejunal loops encased within the fibrocollagenous membrane, consistent with the abnormality seen on preoperative CT. No discrete cystic lesion, tumor, internal hernia, or mesenteric defect was identified. All bowel appeared viable, with no evidence of necrosis or perforation. Given the absence of non-viable bowel, no resection was required.

The abdominal cavity was irrigated, and the laparotomy incision was closed in layers. Estimated blood loss was approximately 100 mL.

A nasogastric tube was left in situ for postoperative decompression, and the patient was transferred to the high-dependency unit for ongoing monitoring.

Histopathology

Multiple fragments of cream-grey tissue were submitted as “encapsulating membrane,” measuring up to 40 × 20 × 3 mm. Microscopic examination demonstrated a hyalinized fibrous tissue capsule with areas of hemorrhage and congestion. A mild acute inflammatory infiltrate was present. No granulomas were identified, and there was no evidence of malignancy. These findings supported a diagnosis of non-granulomatous fibrosing encapsulating peritonitis, in keeping with SEP.

Ziehl-Neelsen staining for acid-fast bacilli and IgG4 immunohistochemistry were not performed on the histopathology specimen. Therefore, while the absence of granulomas and malignancy, a negative QuantiFERON-TB ELISA, and multidisciplinary microbiology and respiratory review made tuberculous peritonitis and malignancy unlikely on the available evidence, the lack of dedicated tissue mycobacterial testing and IgG4 immunohistochemistry is acknowledged as a limitation.

Follow-up and outcomes

The patient remained hemodynamically stable following surgery, and a nasogastric tube was maintained postoperatively for decompression (Table 1). On postoperative day 12, the nasogastric tube was dislodged during an episode of vomiting and was subsequently reinserted on postoperative day 13. He thereafter declined further nasogastric tube reinsertion despite ongoing bilious vomiting, which persisted until endoscopic decompression. Repeat contrast-enhanced CT was performed on postoperative day 13 in response to persistent bilious vomiting and rising inflammatory markers. At that point, the total leukocyte count was 12.1 × 10⁹/L, and CRP was 172 mg/L, consistent with an ongoing inflammatory response. Albumin decreased to 32 g/L, likely reflecting a combination of acute inflammation, physiological stress, and reduced nutritional intake. CT demonstrated a short-segment DJ intussusception with proximal duodenal dilatation, associated mesenteric stranding, and a small pelvic fluid collection, without free intraperitoneal gas or evidence of bowel compromise (Figure 2). No intussusception had been identified at the index operation, and this was therefore considered an early postoperative complication rather than a pre-existing finding. A PICC line was inserted on postoperative day 15, and total parenteral nutrition was commenced to support ongoing nutritional requirements while oral intake remained limited.

**Table 1:** Formal timeline of events CT: computed tomography, ELISA: enzyme-linked immunosorbent assay, PICC: peripherally inserted central catheter.

Timing	Event/outcome
Day of admission	Emergency department presentation, with 7-8 weeks of abdominal pain, bilious vomiting, constipation, and weight loss. Nasogastric decompression, intravenous fluids, proton pump inhibitor therapy, and CT imaging were initiated
Postoperative day 0	Emergency laparotomy was performed, with division/release of encapsulating membrane and adhesiolysis. The bowel was viable, and no resection was required
Postoperative day 2	QuantiFERON-TB ELISA was negative, and microbiology review concluded that tuberculosis was unlikely
Postoperative day 6	Ongoing postoperative recovery. Onset of persistent bilious vomiting despite nasogastric decompression
Postoperative day 12	The nasogastric tube was dislodged/expelled during vomiting. Bilious vomiting persisted
Postoperative day 13	Repeat contrast-enhanced CT demonstrated short-segment duodenojejunal intussusception, with proximal duodenal dilatation, mesenteric stranding, and a small pelvic collection, without free gas or bowel compromise
Postoperative day 15	A PICC line was inserted, and total parenteral nutrition commenced because oral intake remained limited
Postoperative day 22	Esophagogastroduodenoscopy with pneumatic decompression was performed
Postoperative day 26	Total parenteral nutrition was discontinued; patient tolerating oral intake
Postoperative day 30	Discharged in good condition on a soft diet with safety-netting advice; total hospital stay was approximately 32 days from admission

**Figure 2:**
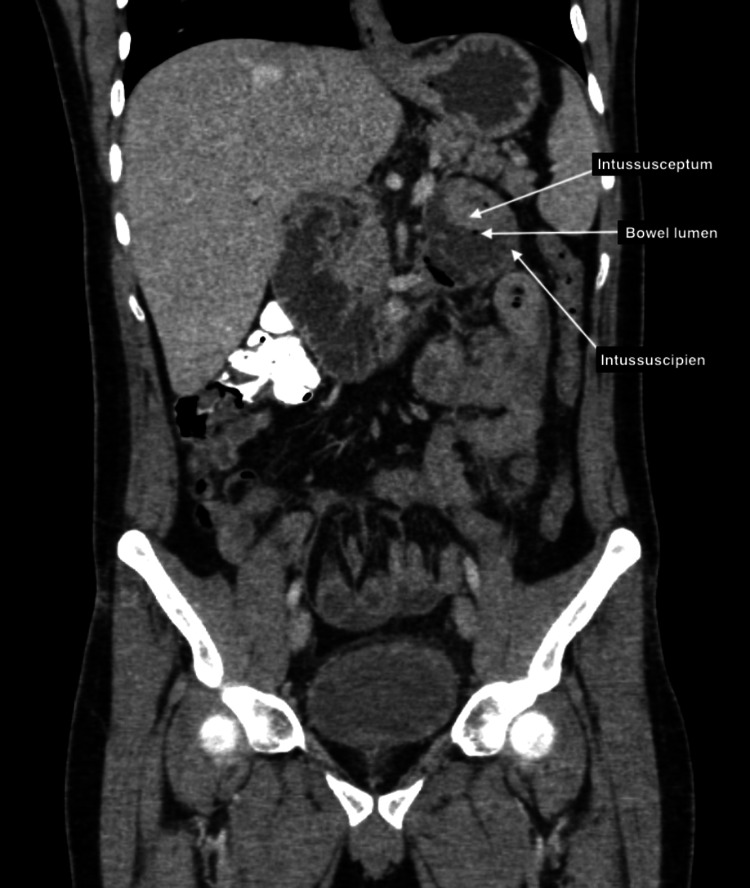
Postoperative computed tomography images There is a persistent short-segment intussusception at the duodenojejunal junction, with secondary proximal duodenal dilatation. The remaining small and large bowels are of normal caliber.

Two management options were considered by the upper gastrointestinal surgical team: endoscopic pneumatic decompression via esophagogastroduodenoscopy or relook laparotomy with further adhesiolysis. Given the patient’s hemodynamic stability, improving nutritional status on parenteral nutrition, and the absence of imaging features of bowel compromise, the team recommended an initial endoscopic approach, incorporating the patient’s strong preference to avoid further surgery, with relook laparotomy reserved for failure or clinical deterioration.

Esophagogastroduodenoscopy was subsequently performed using a Pentax i20-EC34L slim colonoscope (PENTAX Medical, Tokyo, Japan), selected because its extended working length and flexibility allow its use as an enteroscope when deeper small-bowel access is required. The scope was carefully advanced across the DJ flexure using torque control and gradual insufflation, achieving intubation of the jejunum approximately 20 cm beyond the DJ flexure. Severe esophagitis and a large volume of bile were noted in the stomach. No intraluminal pathology was identified within the visualized small bowel; however, a functional transition point was identified at the presumed DJ flexure (Figure 3). Advancement beyond this point appeared to straighten the residual kink and facilitate bile passage, consistent with endoscopic decompression.

**Figure 3:**
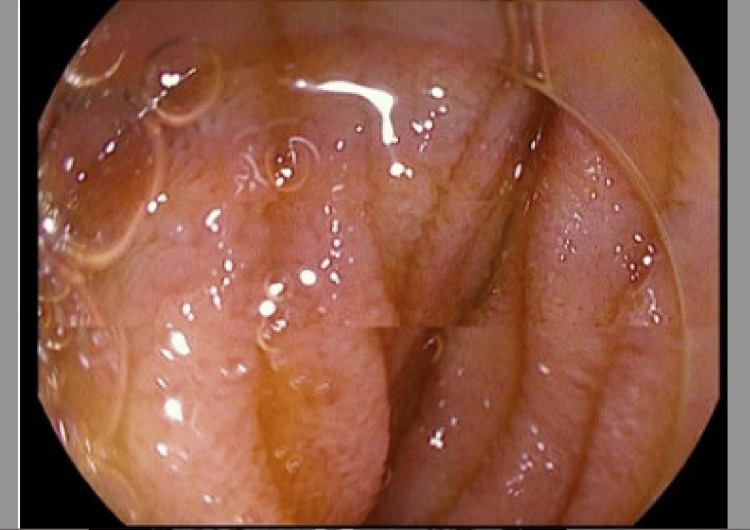
Endoscopic image of the fourth part of the duodenum demonstrating a functional transition point at the duodenojejunal flexure Once this area was traversed, bile appeared to pass more freely distally.

Following discharge, the patient was discussed at the upper gastrointestinal multidisciplinary team meeting, where a review of all investigations confirmed no evidence of malignancy, and he was discharged from the surveillance pathway. Interval high-resolution chest CT demonstrated significant improvement in the previously noted right-sided changes, with only mild residual ground-glass nodularity consistent with a resolving inflammatory or infective process. No new or suspicious pulmonary lesions, pleural effusion, or intrathoracic lymphadenopathy were identified, and he was subsequently discharged from respiratory follow-up. At 13 weeks post-discharge, telephone follow-up via next of kin confirmed no recurrent vomiting or obstructive symptoms, and he was reported to be tolerating oral intake and had returned to baseline activity. At 28 weeks post-discharge, email correspondence with the patient confirmed that he was abroad. He reported occasional mild postprandial abdominal discomfort but denied frank vomiting, obstructive symptoms, or significant functional impairment. No formal in-person surgical follow-up had been arranged prior to his departure, and in-person review has not been possible since.

## Discussion

This case describes a primary SEP presenting as recurrent subacute obstruction progressing to high-grade proximal small bowel obstruction. The case is notable for three reasons. First, the disease was focal and proximal, centered around the DJ flexure and proximal jejunum, where CT appearances were initially interpreted as an internal hernia or a cystic jejunal abnormality. Second, a definitive diagnosis was made intraoperatively through the identification of an extrinsic fibrocollagenous membrane encasing the small bowel, with histopathology supporting non-granulomatous encapsulating peritonitis. Third, the postoperative course was complicated by short-segment DJ intussusception, managed through a multidisciplinary strategy incorporating nutritional optimization, decompression, endoscopic intervention, and a contingency plan for relook laparotomy.

No major secondary cause was identified. The patient had no history of peritoneal dialysis, prior abdominal surgery, chronic liver disease, malignancy, or known autoimmune disease. QuantiFERON-TB ELISA was negative, autoimmune and viral screening did not identify an active systemic inflammatory or infective cause, and histology demonstrated hyalinized fibrous tissue without granulomas or malignancy. On the available clinical, radiological, serological, and histopathological evidence, the case was therefore considered most consistent with primary SEP. This distinction is important because much of the broader literature on encapsulating peritoneal disease relates to peritoneal dialysis-associated encapsulating peritoneal sclerosis, where cohort data, prognosis, and management guidance are more developed but not directly generalizable to idiopathic SEP [3,7-11].

SEP remains rare, and the evidence base for primary disease is derived predominantly from case reports, small series, and narrative reviews [1-5,12-14]. In patients without a history of peritoneal dialysis, SEP has been reported across a broad age range; in a review of 118 cases, the mean age was 39 years [14]. The literature confirms that presentation is heterogeneous. One report described multiple recurrent episodes of bowel obstruction over several years, repeatedly resolving with conservative management before laparotomy established the diagnosis [15]. Another described constipation, vomiting, and abdominal pain culminating in acute bowel occlusion [16]. Elsewhere, patients have presented with complete small bowel obstruction and marked weight loss, prompting urgent surgical exploration [17]. Other reports describe more slowly progressive colicky pain, nausea/vomiting, constipation, and weight loss over several months [12,18]. Across broader syntheses of published cases, abdominal pain is the predominant symptom, although distension, vomiting, and an abdominal mass are also common [14].

Our patient’s course reflected this relapsing pattern, with recurrent obstructive episodes preceding definitive operative diagnosis. Symptom recurrence should therefore not be interpreted as reassuring. When episodes repeatedly converge on obstructive physiology without a durable alternative diagnosis, clinicians should reconsider rarer extrinsic causes, such as SEP, particularly in patients without prior abdominal surgery. Repeated self-limiting presentations may delay diagnosis because symptoms can be attributed to gastroenteritis, functional gastrointestinal disorders, adhesive-type obstruction, or inflammatory bowel disease.

Contrast-enhanced CT is considered the most informative preoperative imaging modality for SEP, but characteristic findings are not consistently present [3-6,13]. Typical features include clustered small bowel loops within a sac-like membrane, peritoneal thickening or enhancement, and variable ascites or calcification [3-6,13,14]. However, focal disease may not produce the classic “cocoon” or “cauliflower” configuration. Several reports illustrate that proximal disease can be misinterpreted. One report described CT findings interpreted as internal herniation at the DJ flexure, yet laparotomy demonstrated SEP without a hernia defect [5]. Another described SEP presenting as an internal hernia mimic, with diagnosis confirmed surgically [17].

In our patient, CT findings were dominated by a proximal jejunal transition point with upstream gastric and duodenal dilatation and an apparent low-attenuation cystic abnormality. In retrospect, the most coherent explanation consistent with the operative findings is that the apparent “collection” represented clustered, encapsulated jejunal loops contained within the fibrocollagenous membrane, rather than a discrete cystic lesion or internal hernia. Therefore, when CT is interpreted as an internal hernia without a clear hernia configuration, or when recurrent unexplained obstruction occurs in a patient without prior surgery, targeted radiological review should assess for loop clustering, membrane-like encapsulation, subtle peritoneal thickening, and absence of a true mesenteric defect [2-6,13,17].

No validated blood marker reliably diagnoses SEP, and routine laboratory abnormalities are generally nonspecific [3-5,13,14]. In encapsulating peritoneal disease research, inflammatory and mesothelial markers, including IL-6 and CA-125, have been explored, particularly in dialysis-associated disease, but these do not form part of a routine diagnostic algorithm [3,5,7-11,13,14]. Some reports have proposed immune-mediated mechanisms, including IgG4-related disease. IgG4-related SEP has been described in isolated cases, including reports demonstrating IgG4-positive tissue findings and inflammatory marker abnormalities [5,19]. These observations support mechanistic heterogeneity, but they do not justify applying immune-mediated labels to all SEP. In the present case, IgG4 serology, cytokine profiling, and IgG4 immunohistochemistry were not performed. There were no recorded systemic, radiological, or histological features strongly suggestive of IgG4-related disease. Discussion of immune overlap should therefore remain cautious and framed as an area of uncertainty rather than a patient-specific conclusion.

Histopathology is supportive rather than independently diagnostic, but it is important for excluding secondary causes and mimics. Typical findings include fibrocollagenous or fibrotic tissue with variable inflammatory infiltrate; granulomas or malignant cells should prompt evaluation for alternative etiologies such as tuberculosis or peritoneal carcinomatosis [1-5,13,14]. In this case, histology demonstrated a hyalinized fibrous tissue capsule with hemorrhage, congestion, and mild acute inflammatory change, without granulomas or malignancy. Together with negative QuantiFERON-TB ELISA, absence of lymphadenopathy on CT, and multidisciplinary microbiology and respiratory review, this supported classification as primary non-granulomatous SEP on the available evidence. However, tissue Ziehl-Neelsen staining, acid-fast bacilli testing, and dedicated mycobacterial culture were not performed on the membrane specimen and would have strengthened microbiological exclusion of tuberculous peritonitis.

Management of SEP is guided by symptom severity, disease extent, and suspected etiology. Conservative strategies, including nutritional optimization and selected medical therapies such as corticosteroids or tamoxifen, have been described mainly in secondary encapsulating peritoneal sclerosis and other selected contexts, but the evidence remains limited and is not directly generalizable to idiopathic obstructive SEP [3,5,11,12,20]. In patients with high-grade obstruction, the operative cornerstone is membrane division with adhesiolysis to restore bowel mobility and relieve extrinsic kinking [1-5,13,15]. Bowel resection should be avoided unless viability is clearly compromised because SEP is predominantly an extrinsic constrictive process, and unnecessary resection risks avoidable morbidity, including short bowel syndrome [1-5,13,14]. This case aligns with these principles. The encapsulating membrane was divided, the small bowel was released, and no resection was required because the bowel remained viable and no intrinsic tumor, perforation, mesenteric defect, or internal hernia was identified.

Postoperative complications following surgical release of SEP are well recognized and should be anticipated. Reported early complications include postoperative small bowel obstruction, intra-abdominal sepsis or infection, serosal tears with perforation, and enterocutaneous fistula formation, while later complications include recurrent adhesive obstruction; short bowel syndrome is primarily a risk where bowel resection is undertaken [1,3-5,12-15]. Although long-term recurrence of the “cocoon” itself appears uncommon in most series, morbidity and mortality have been reported, particularly in secondary encapsulating peritoneal sclerosis and in patients with significant comorbidity, with deaths most commonly related to sepsis and multiorgan failure [3,8-10,13].

Postoperative intussusception is a recognized but underappreciated cause of early postoperative bowel obstruction following abdominal surgery. Historical series and isolated adult case reports have described intussusception presenting as postoperative obstruction after laparotomy or other abdominal procedures [21-23]. These reports are not specific to surgery for small bowel obstruction or SEP and instead arise in varied postoperative settings. Nevertheless, they emphasize that recurrent obstruction following surgery should not be attributed solely to ileus or adhesions, and that alternative mechanical causes should also be considered. Evidence describing intussusception specifically after laparotomy for bowel obstruction, or following surgical release of encapsulating peritoneal disease, remains extremely limited.

Endoscopic management has been described in selected intussusception cases, including rare adult and postoperative presentations [24-26]. These reports do not describe intussusception arising after surgery for SEP or bowel obstruction laparotomy, but they support the broader principle that minimally invasive intervention may be appropriate in carefully selected, clinically stable patients when there is no imaging or clinical evidence of bowel compromise. In our case, persistent postoperative obstruction reflected a discrete mechanical phenomenon, namely, short-segment DJ intussusception, rather than diffuse re-encapsulation. This supported a structured approach prioritizing exclusion of bowel compromise, nutritional optimization, decompression, and endoscopic management, with relook laparotomy reserved for clinical deterioration or failure of minimally invasive treatment. To our knowledge, there are no published reports describing endoscopic management of postoperative DJ intussusception specifically complicating SEP release or laparotomy for bowel obstruction. This case therefore represents a novel application of endoscopic decompression in this context.

The key practical implication is not that endoscopy should replace surgery, but that in a stable patient with focal postoperative obstruction and no evidence of ischemia, perforation, or peritonitis, multidisciplinary endoscopic intervention may be considered part of a clearly defined escalation pathway.

Limitations

Although admission laboratory data and postoperative inflammatory markers were available, laboratory assessment was not comprehensive for all potential secondary or immune-mediated causes of SEP. In particular, serum IgG4, cytokine profiling such as interleukin-6, and IgG4 immunohistochemistry of the membrane specimen were not performed; therefore, an immune-mediated or IgG4-related overlap cannot be definitively excluded. Tuberculosis was considered because of the patient’s remote residence in a tuberculosis-endemic region. QuantiFERON-TB ELISA was negative, CT demonstrated no lymphadenopathy, histology showed no granulomatous inflammation, and multidisciplinary microbiology and respiratory review considered tuberculosis unlikely. On this basis, tissue Ziehl-Neelsen staining and acid-fast bacilli testing were not performed. However, no dedicated mycobacterial staining or culture was performed on the membrane specimen. This could have provided additional microbiological confirmation and would have strengthened the exclusion of tuberculous peritonitis as a secondary cause. Follow-up was limited to remote telephone and email correspondence, without long-term in-person clinical review, repeat abdominal imaging, or objective nutritional reassessment. Therefore, late recurrence, adhesive obstruction, weight recovery, and the durability of endoscopic management could not be fully assessed. As a single case report, these findings should be interpreted as hypothesis-generating rather than definitive guidance for the management of postoperative DJ intussusception following SEP release.

Patient perspective

Written informed consent was obtained from the patient for publication of this case report and accompanying anonymized clinical images.

Ethical approval

Formal research ethics committee approval was not required for this single-patient case report, as it describes routine clinical care and did not involve any experimental intervention. Written patient consent for publication was obtained.

## Conclusions

In conclusion, this case highlights primary SEP as a rare but important cause of recurrent, unexplained small bowel obstruction in patients without prior abdominal surgery. Focal involvement at the DJ flexure can mimic an internal hernia or a cystic jejunal lesion on CT, and definitive diagnosis may only be established intraoperatively. Management should prioritize careful membrane division and adhesiolysis with preservation of viable bowel. Persistent postoperative obstruction should prompt reassessment for discrete mechanical complications, including intussusception, rather than being attributed automatically to ileus, adhesions, or recurrent encapsulation. In selected clinically stable patients, multidisciplinary decision-making may support minimally invasive endoscopic decompression, with relook laparotomy reserved for clinical deterioration or failure of conservative or endoscopic management.
